# Skilled delivery service utilization and its association with the establishment of Women’s Health Development Army in Yeky district, South West Ethiopia: a multilevel analysis

**DOI:** 10.1186/s13104-018-3140-0

**Published:** 2018-01-30

**Authors:** Melese Girmaye Negero, Yifru Berhan Mitike, Abebaw Gebeyehu Worku, Tafesse Lamaro Abota

**Affiliations:** 1 Department of Public Health, College of Medical and Health Sciences, Wollega University, Nekemte, Ethiopia; 2 Federal Democratic Republic of Ethiopia, Ministry of Health, Addis Ababa, Ethiopia; 3 Amhara National Regional State, Health Bureau, Bahirdar, Ethiopia; 4 Department of Nursing, College of Health Sciences, Mizan-Tepi University, Mizan, Ethiopia

**Keywords:** Community-based, Ethiopia, Multilevel analysis, Skilled delivery care, Women’s Health Development Army

## Abstract

**Background:**

Because of the unacceptably high maternal and perinatal morbidity and mortality, the government of Ethiopia has established health extension program with a community-based network involving health extension workers (HEWs) and a community level women organization which is known as “Women’s Health Development Army” (WHDA). Currently, the HEWs and WHDA network is the approach preferred by the government to register pregnant women and encourage them to link in the healthcare system. However, its association with skilled delivery service utilization is not well known.

**Methods:**

A community-based cross-sectional study was conducted from January to February 2015. Within 380 clusters of WHDA, a total of 748 reproductive-age women who gave birth in 1 year preceding the study, were included using multistage sampling technique. The data were entered into EPI info version 7 statistical software and exported to STATA version 11 for analysis. Multilevel analysis technique was applied to check for an association of selected variables with a utilization of skilled delivery service.

**Results:**

About 45% of women have received skilled delivery care. A significant heterogeneity was observed between “Women’s Health Development Teams (clusters)” for skilled delivery care service utilization which explains about 62% of the total variation. Individual-level predictors including urban residence [AOR (95% CI) 35.10 (4.62, 266.52)], previous exposure of complications [AOR (95% CI) 3.81 (1.60, 9.08)], at least four ANC visits [AOR (95% CI) 7.44 (1.48, 37.42)] and preference of skilled personnel [AOR (95% CI) 8.11 (2.61, 25.15)] were significantly associated with skilled delivery service use. Among cluster level variables, the distance of clusters within 2 km radius from the nearest health facility was significantly associated [AOR (95% CI) 6.03 (1.92, 18.93)] with skilled delivery service utilization.

**Conclusions:**

In this study, significant variation among clusters of WHDA was observed. Both individual and cluster level variables were identified to predict skilled delivery service utilization. Encouraging women to have frequent ANC visits (− 4 and above), enhancing awareness creation towards the delivery care attendance, constructing more health facilities and roads in hard to reach areas and establishing telemedicine services are recommended.

## Background

As World Health Organization (WHO) defined, skilled care at delivery is a delivery service provided by “an accredited health professional such as a midwife, doctor or other nurse-who have been educated and trained to proficiency in the skills needed to manage normal (uncomplicated) pregnancies, childbirth and the immediate postnatal period, and in the identification, management and referral of complications in women and newborns” [1–3]. An estimated 74% of maternal deaths could be prevented if all women had access to skilled delivery and emergency obstetric care services [4–6]. Despite the existence of proven interventions to prevent disability or death during pregnancy and childbirth; maternal and perinatal mortality still remains to be major public health problems among sub-Saharan African countries, including Ethiopia [2, 6–11]. The WHO has estimated that 289,000 women died during pregnancy and childbirth in 2013 globally, a majority of which were in developing countries with limited access to skilled maternity services [12, 13]. According to Ethiopian Demographic and Health Survey (EDHS) 2011 and International Monetary Fund (IMF) consultation-staff report of 2014, the maternal mortality ratios in Ethiopia were 676/100,000 and 420/100,000 live births, respectively, which are still among the highest in the world and sub-Saharan African Countries [14, 15]. Skilled personnel attended delivery care is a well known and proven intervention to reduce maternal and perinatal mortality and morbidity [1, 10, 11, 16–23]. The unacceptably high maternal and perinatal mortalities in Ethiopia could probably be due to the very low coverage of skilled delivery care in the country. The EDHS 2011 and mini EDHS 2014 reported that only 10 and 15% of pregnant women in Ethiopia, respectively, received skilled delivery care which is among the lowest in the world and sub-Saharan African Countries [14, 24]. According to the-EDHS 2011, the reasons for not attending health facilities for delivery care were: “it was not important (61%)”, “not familiar (30%)”, and “health facilities were geographically inaccessible (14%)”, respectively, while the mini-EDHS 2014 revealed that similar reasons were given by 45, 33 and 22%, respectively of women who didn’t give their last birth at health facilities [14, 24].

This indicates that lack of awareness, poor attitude, and inaccessibility of health facilities were the major factors for low coverage of skilled personnel attended delivery and hence high proportion of maternal and perinatal mortalities in Ethiopia.

To have effective community-based programs, the health extension program of Ethiopia involved community level women organization which is known as “Women’s Health Development Army (WHDA)” Since 2011. The WHDA is Women’s Health Development Team (WHDT) organized as one-to-five (about 5–6 members and one of them is the leader) and one-to-thirty (further grouping of the 6 sub-groups involving 30 members) of women in the nearby households. The one-to-thirty development team leaders are reporting the lists of pregnant women to the health extension worker (HEW) and the HEW send it to the Health Center (HC) regularly. At HC, the expected date of delivery (EDD) of all listed pregnant women will be calculated and midwives try different mechanisms to trace based on their EDD [25, 26].

Currently, the HEWs and WHDA network is believed to be the best approach to implement health extension package (HEP) activities like enhancing women’s seeking behavior in attending skilled maternity services [25, 26]. However, no or only little is known about the association of skilled delivery care service utilization with the establishment of this army. Therefore, this study was planned to assess skilled delivery care service utilization and associated factors at individual and WHDT levels in Yeky district, Southwest Ethiopia.

## Methods

### Study design and setting

A community-based cross-sectional study was conducted over 2 months (January–February 2015) among women of reproductive age who gave birth 1 year preceding the study at Yeky district, Sheka Zone, Southwest Ethiopia. The district is located about 562 km from the capital, Addis Ababa. It is structured into 22 rural “kebeles” (the lowest administrative units with a size of about 1000 households) and one town administration, Tepi town. Tepi is structured into three administrative kebeles. There are about 845 established and functioning WHDTs in the district, 632 in the rural kebeles and 213 in the district center, Tepi.

In 2014, there were an estimated 172,520 people in Yeky district of which reproductive-aged women comprised 39,680. Majority of the inhabitants (96%) are rural dwellers. Agriculture is the major means of livelihood. The district is also well known for its cash crops, like coffee and spices. The district has a total of 30 functioning public health institutions (22 Health posts, 7 health centers, and 1 district hospital). Local FM radios newly opened in the area are also playing some roles in creating awareness about the importance of delivering at health facilities.

### Study population and sampling

Those women of reproductive age groups who gave birth in 1 year preceding the study in the randomly selected seven kebeles the in the study irrespective of their birth outcome. The sample size was calculated using single population proportion formula and Epi-Info version-7 statistical software based on the following assumptions: margin of error of 3%, the design effect of 2, non response rate of 10%, a confidence level of 95%, and proportion for skilled delivery service of 9.4% [24]. Accordingly, the total sample size was 800. We have tested the null hypothesis that there was no variation in skilled delivery care service utilization between clusters or WHDTs using multilevel analysis.

Cluster sampling was applied to select the study population.

The kebeles with their WHDT (clusters) were selected randomly. Accordingly, 7 (5 rural and 2 urban) kebeles with a total of 380 WHDT were selected.

### Data collection and analysis

A house to house interview was done using structured and pretested questioner. The questioners were employed to obtain information on socio-demographic, accessibility, perceived needs (benefits), and WHDT related characteristics. A total of 35 qualified data collectors and 14 supervisors (5 data collectors and 2 supervisors for each kebele) were deployed.

In this study, skilled delivery care service utilization refers utilization of delivery care service by mothers at a health center, hospital or private clinic for their last birth.

During analysis, performance of WHDTs was classified as best or grade “A” if all households have achieved 91–100% of all the seventeen HEPs, including giving birth at health center, hospital or private clinics for eligible women, good or grade “B” if all households have achieved 81–90% of all the seventeen HEPs, and poor or “C” if all households have achieved < 80% of all the seventeen HEPs.

Health extension package refers to a list of mainly preventive and promotive activities to be performed through the collaboration between HEWs and the local community [25, 26]. It is categorized into four pillars of activities, namely family health, hygiene and sanitation, communicable disease control and health education and communication. Family health activities include immunization, family planning, maternal and child health care (e.g. skilled delivery utilization), preparation and utilization of balanced food and elimination of harmful traditional practices. Hygiene and sanitation activities include latrine construction and utilization, food hygiene, solid and liquid waste management, water sanitation, personal hygiene, home management and pest and vector control, while communicable disease control activities include prevention and control of malaria, HIV/AIDS and TBC. Providing first aid and integrated community case management of common childhood illnesses are also among the HEP activities [27, 28].

Similarly, self-sustained WHDT refers to availability of enough production (food or cash crops) for feeding population in the village (WHDT).

The data was entered using EPI Info version 7 statistical software and was exported to STATA version 11 for analysis. The predictors of skilled delivery care service utilization were assessed using the multilevel binary logistic regression analysis technique to measure individual and group-level (WHDT) variables.

## Results

### Socio-demographic and obstetric characteristics of the study participants

A total of 748 women aged 15–49 years participated in the study. The majority (84.5%) were rural dwellers. Most (95.6%) were currently married. The mean (± SD) age of the participants was 25.8 (± 5.3) where the majority (32.9%) were between 25–29 years category and 6.7% were teenagers (< 20 years). Nearly two-thirds (62.3%) were Protestant Christians and about 92% were housewives. About 60% of the respondent’s households had about 10 USD or less average monthly income while only 11% could get more than about 50 USD per month. Regarding the educational level, 45.7% didn’t attend any formal education (Table 1).

**Table 1:** Selected socio-demographic individual-level characteristics of women who gave birth in 1 year preceding the study, Yeky district, 2015, (N = 748)

Background characteristics	Category	Number (%)
Residence	Urban	116 (15.5)
	Rural	632 (84.5)
Current age in years	16–19	50 (6.7)
	20–24	241 (32.2)
	25–29	246 (32.9)
	30–34	121 (16.2)
	≥ 35	90 (12)
Ethnicity	Keffa	206 (27.5)
	Amhara	143 (19.1)
	Bench	134 (17.9)
	Sheka	95 (12.7)
	Menja	70 (9.4)
	Sheko	34 (4.5)
	Mejengir	18 (2.4)
	Dawuro	18 (2.4)
	Others	30 (4)
Religion	Protestant	466 (62.3)
	Orthodox	183 (24.5)
	Muslim	92 (12.3)
	Others	7 (0.9)
Marital status	Single	18 (2.4)
	Married	715 (95.6)
	Divorced	15 (2)
Husband education (n = 715)	No formal education	188 (26.3)
	Primary education (1–8)	451 (63.1)
	Secondary education and above	76 (10.6)
Household monthly average income in birr	≤ 200	448 (59.9)
	201–1000	217 (29)
	> 1000	83 (11.1)
Who usually makes decisions on the mother’s healthcare? (Autonomy)	Woman ± husband	251 (33.6)
	Husband ± others	497 (66.4)
Age when married first in years (n = 736)	≤ 15	137 (18.6)
	16–19	420 (57.1)
	≥ 20	179 (24.3)
Parity	1	224 (29.9)
	2–4	403 (53.9)
	5 and above	121 (16.2)
Maternal education	No formal education	342 (45.7)
	Primary education (1–8)	367 (49.1)
	Secondary education and above	39 (5.2)
Listen to radio	Yes	259 (34.6)
	No	489 (65.4)

Three hundred fifty (46.8%) mothers were teenagers and 16 (2.1%) were 35 and above years when they gave birth to their first baby. The mean (± SD) age at the first birth was 19.9 (± 2.6) years. The majority (53.9%) of respondents had parity between 2–4 births (Table 2).

**Table 2:** Utilization of skilled delivery care service using selected individual and group level characteristics in Yeky district, 2015

Characteristics	Category	N	Number (percent) of women reporting skilled delivery care service use
Residence	Urban	116	111 (95.7)
	Rural	632	223 (35.3)
Maternal education	Formal education	406	223 (54.9)
	No formal education	342	111 (32.5)
Husband education	Formal education	527	260 (49.3)
	No formal education	188	55 (29.3)
Household monthly average income in birr	≤ 200	448	145 (32.4)
	201–1000	217	118 (54.4)
	> 1000	83	71 (85.5)
Woman’s autonomy in her own health care	Woman ± husband	251	150 (59.8)
	Husband ± others	497	184 (37)
ANC follow up for the last birth	No	83	7 (8.4)
	1–3	229	106 (46.3)
	≥ 4	436	221 (50.7)
ANC in previous pregnancy	Yes	359	146 (40.7)
	No	165	47 (28.5)
Ever faced complications during previous pregnancies or childbirth	Yes	96	48 (50)
	No	428	146 (34.1)
Women preferred skilled personnel for delivery care	Yes	513	314 (61.2)
	No	235	20 (8.5)
Awareness of places to get skilled providers	Yes	518	303 (58.5)
	No	230	31 (13.5)
Listen to radio	Yes	259	169 (65.3)
	No	489	165 (33.7)
Average distance of WHDT from the nearest HF with skilled care (KMs)	≤ 2	213	168 (78.9)
	3–5	230	80 (34.8)
	> 5	305	86 (28.2)
Self-sustained WHDT	Yes	592	280 (47.3)
	No	156	54 (34.6)
Performance level of WHDT	Best performing	302	128 (42.4)
	Good performing	374	166 (44.4)
	Poor performing	72	40 (55.6)
	Total	748	334 (44.7)

### Delivery practice

More than half (51.6%) of respondents had given their last birth at home. Skilled delivery care was received by 334 (44.7%) respondents for their recent birth. Regarding the birth attendants, 298 (39.8%), 292 (39%), 54 (7.2%), 44 (5.9%), 35 (4.7%) and 25 (3.3%) respondents were attended by their relatives, nurses, self (no one), doctor, health extension workers (HEWs) and traditional birth attendants for their last birth, respectively. Twenty-seven (3.6%) respondents had delivered by cesarean section. Regarding their reasons for seeking health facility delivery, 322 (89%), 296 (81.8%), 194 (53.6%), and 19 (2.5%) respondents reported that it was for the maternal wellbeing, for the fetal wellbeing, to control bleeding and to prevent retained placenta, respectively.

For the respondents who did not give their last birth at health facilities, 238 (61.7%), 55 (14.2%) and 52 (13.5%) respondents reported that it was because of easy labour, lack of transport and health facility was too far, respectively, while 42 (10.9%), 18 (4.7%) and 17 (2.3%) respondents reported that it was because of lack of awareness, overnight labour, and birth delivered on the road to health facilities, respectively. Twelve (1.6%) respondents reported that it was not accustomed to delivering at health facilities (Table 2).

### Multilevel (mixed effects) logistic regression analysis

The multilevel analysis was started from the intercept only (null) model and then finalized on the random intercept model. The results depicted in Table 3 showed that there was significant heterogeneity between WHDTs with regard to skilled delivery service utilization. The intracluster correlation coefficient (ICC) in the null model for skilled delivery service utilization was 61.72%. In other words, 61.72% of the variation in utilization of skilled delivery care services between the WHDTs is due to the differences between the WHDTs (between-cluster variation) (Table 3).

**Table 3:** Parameter coefficients of the intercept only (null) model in using skilled delivery care service, Yeky district, 2015

Skilled delivery care service	
Level 2 variance: VAR (_cons)	5.304* (3.209, 8.768)
Intracluster correlation coefficient (Rho)	0.6172
Deviance (− 2 log likelihood)	864

* Significant

The mixed effect modeling indicated that the individual level characters including urban residence, previous exposure of complications, at least four ANC visits and preference of skilled personnel were significantly associated with skilled delivery service utilization. For instance, women residing in urban were 35.10 times (95% CI 4.62, 266.52) more likely to utilize skilled delivery care services as compared to their counterparts in the rural area.

Similarly, women who had four and above ANC visits were 7.44 times (95% CI 1.48, 37.42) more likely to use skilled delivery service as compared those women who did not have any ANC follow up visits for the pregnancy of their last birth. The odds of utilizing skilled delivery service was 3.81 times (95% CI 1.60, 9.08) more in women who experienced at least one complication during their previous pregnancies or childbirths as compared to those women who did not face any complications during their previous pregnancies or childbirth.

Among the level 2 (WHDT) variables, the distance of WHDT within 2 km radius from the nearest health center or hospital showed positive and significant association with skilled delivery service utilization (Table 4).

**Table 4:** Bivariate and multivariate multilevel analysis by predictors of skilled delivery care service utilization, Yeky district, 2015

Characteristics	N	Crude OR (95% CI)	Adjusted OR (95% CI)	P value (AOR)
Individual characters				
Residence				
Urban	116	109.44 (28.70, 417.29)	35.10 (4.62, 266.52)	0.001
Rural	632	1	1	
Maternal education				
Formal education	406	2.18 (1.38, 3.46)	1.04 (0.51, 2.12)	0.919
No formal education	342	1	1	
Husband education				
Formal education	527	2.17 (1.27, 3.69)	1.29 (0.61, 2.75)	0.503
No formal education	188	1	1	
Woman’s autonomy on her own healthcare				
Woman ± husband	251	1.66 (0.94, 2.92)	2.15 (0.94, 4.96)	0.071
Husband ± others	497	1	1	
ANC follow up for the last birth				
No	83	1	1	0.353
1–3	229	14.42 (4.55, 45.74)	2.22 (0.41, 11.90)	0.015
≥ 4	436	24.05 (7.76, 74.52)	7.44 (1.48, 37.42)	
^a^ANC follow up for previous pregnancy				
Yes	359	2.77 (1.38, 5.55)	1.57 (0.67, 3.68)	0.300
No	165	1		
Complications in previous pregnancies or births				
Yes	96	2.98 (1.42, 6.25)	3.81 (1.60, 9.08)	0.003
No	428	1	1	
Skilled personnel preferred for delivery care				
Yes	513	38.37 (17.03, 86.43)	8.11 (2.61, 25.15)	0.000
No	235	1	1	
Awareness of places to get skilled providers for delivery care				
Yes	518	10.41 (5.68, 19.07)	3.00 (1, 9.00)	0.050
No	230	1	1	
Listen to radio				
Yes	259	3.83 (2.27, 6.48)	0.82 (0.36, 1.86)	0.638
No	489	1	1	
WHDT characteristics				
Average distance of WHDT from the nearest HF with skilled care (KMs)				
≤ 2	213	15.62 (7.21, 33.86)	6.03 (1.92, 18.93)	0.002
3–5	230	1.46 (0.76, 2.81)	1.34 (0.54, 3.31)	0.522
> 5	305	1	1	
Self-sustained WHDT				
Yes	592	3.27 (1.16, 9.16)	0.74 (0.24, 2.29)	0.603
No	156	1	1	
The performance level of WHDT				
Best performing	302	0.58 (0.20, 1.69)	2.14 (0.38, 12.27)	0.391
Good performing	374	0.89 (0.32, 2.51)	4.38 (0.75, 25.56)	0.101
Poor performing	72	1	1	
The random part of the model:	*VAR (cons) i.e. level-2 variance*		2.84 (1.17, 6.91)	
	*Rho, intra-class correlation coefficient.*		0.46	
	*Deviance (−* *2 log likelihood), G*^*2*^		402	

^a^Antenatal care follow-up for previous pregnancy just before the last birth

## Discussion

The study showed that utilization of skilled delivery care service was well above the recent EDHS 2011 and mini EDHS 2014 reports of the country and the region, respectively [14, 24].

This could be due to the added number of health facilities with their appropriate health professionals and materials. Five years before the study, there were only two functioning health centers in the study area. Now, there are seven (07) health centers in different areas of the district and one district hospital at the center. Local mass media like FM radios newly opened in the area are also playing some roles in changing the attitude of the local community towards skilled maternity service utilization. All-time accessible roads might have also improved service utilization relative to the previous one.

Our analysis showed that utilization of skilled delivery care service by individual women was dependent on the joint effect of individual and group (WHDT) level factors. The ICC result indicated that the contribution of the unobserved group (WHDT) level characteristics was 61.72% for skilled delivery care service utilization. In the random intercept model, the between-group (WHDT) variance was significant indicating that controlling variables at different levels is important to avoid misleading associations. Past studies based on multilevel analysis indicated similar findings [29–33].

The result consistently depicted that those women who preferred skilled attendants for their delivery care were more likely to utilize skilled delivery care services than those who didn’t prefer skilled attendants.

Women may get the knowledge from previous exposure of the services, through mass media, from community-based health educations or formal education.

This implicates that attitudinal changes towards seeking skilled personnel attended delivery services through repeated and multisectoral community, school and facility-based educations could bring positive effects on skilled delivery service utilization. Our finding was consistent with other studies done elsewhere in the country [29, 34–36].

In the study, the association of urban residence with skilled delivery care service utilization was highly significant. This finding was in agreement with other studies done in the country and other developing countries [2, 6, 16, 37–44]. This could be due to the fact that urban women tend to have better educational, economic and geographic access to health facilities and other promotional activities which are usually urban-centered.

The findings revealed that four and above times antenatal care visit was a positive and significant predictor of skilled delivery service utilization. This finding was consistent with other studies done elsewhere in the country and other sub-Saharan African Countries [2, 20, 40, 45–48]. Antenatal care with skilled providers is one of the proven interventions that reduce maternal mortality by allowing early detection of obstetric complications and giving opportunity in influencing the woman’s decision to utilize skilled delivery care services.

The study also revealed that women who faced any pregnancy or childbirth-related complications in previous time were more likely to seek skilled delivery care services than their counterparts.

This could also be due to the fact that women who faced complications during previous time are more complication sensitive and seek skilled care services for the fear of reoccurring of the complications. This finding is in agreement with other studies done in the country and other developing countries [11, 18, 45, 49–51].

Among the level 2 variables; the distance of WHDT from the nearest health center or hospital determines the utilization of skilled delivery service, consistent with other studies [16, 20, 22, 36, 37, 40, 49, 50, 52]. Distance from health facilities and lack of transport are important deterring factors that hinder mothers from seeking skilled maternity care services, especially delivery care.

This study revealed that there was no statistically significant association between the performance level (activities) of WHDTs and skilled delivery service utilization. This implies that it was not the performance level of WHDTs rather other factors are more relevant to explain the influences at level 2. The finding might also imply the inappropriate system of classifying, evaluating and measuring Women’s Health Development Teams’ performances.

## Conclusion

This study revealed that about 45% of the study subjects have received skilled delivery care service. A significant heterogeneity was observed between “Women’s Health Development Teams (clusters)” for skilled delivery care service utilization. Individual-level predictors including urban residence, previous experience of pregnancy or childbirth-related complications, having four or more ANC visits for the last birth and preference of skilled personnel for care were significantly and independently associated with skilled delivery service utilization.

Although about two-thirds of the variation was explained by cluster difference, only one cluster (WHDT) level character, “distance of WHDTs from the nearest health center or hospital” was identified as the significant predictor of skilled delivery service utilization while the remaining group level characters did not show a significant association.

As a recommendation, community awareness and perception creation towards skilled personnel assisted delivery service utilization need to be enhanced. Encouraging women to have frequent ANC visits (four and above) is also important to enhance skilled delivery service utilization. More health facilities and roads need to be constructed in hard to reach areas. Telemedicine approach where communities get access to affordable on phone health care services could also be beneficial. Implementation and evaluation strategies of “Women’s Health Development Army” need to be revised.

## Limitations

One of the limitations of this study is the difficulty of the respondents in identifying skilled personnel between doctors, midwives, and nurses. Recalling previous events was also the other problem during data collection. Different efforts were done to reduce such errors of categorizing skilled providers and reducing recall bias. With cross-sectional study design, it could be difficult to exhaust all important determinants of skilled delivery care service utilization.

## Abbreviations

ANC: antenatal care
EDHS: Ethiopian Demographic and Health Survey
HEP: health extension program
HEW: health extension workers
ICC: intra-cluster correlation coefficient
WHDA: Women’s Health Development Army
WHO: World Health Organization
WHDT: Women’s Health Development Team
